# Identifying Potential miRNAs–Disease Associations With Probability Matrix Factorization

**DOI:** 10.3389/fgene.2019.01234

**Published:** 2019-12-11

**Authors:** Junlin Xu, Lijun Cai, Bo Liao, Wen Zhu, Peng Wang, Yajie Meng, Jidong Lang, Geng Tian, Jialiang Yang

**Affiliations:** ^1^College of Computer Science and Electronic Engineering, Hunan University, Changsha, China; ^2^Department of Science, Geneis Beijing Co., Ltd., Beijing, China; ^3^School of Mathematics and Statistics, Hainan Normal University, Haikou, China

**Keywords:** diseases, miRNAs, probabilistic matrix factorization, association prediction, receiver operating characteristic curve (ROC)

## Abstract

In recent years, miRNAs have been verified to play an irreplaceable role in biological processes associated with human disease. Discovering potential disease-related miRNAs helps explain the underlying pathogenesis of the disease at the molecular level. Given the high cost and labor intensity of biological experiments, computational predictions will be an indispensable alternative. Therefore, we design a new model called probability matrix factorization (PMFMDA). Specifically, we first integrate miRNA and disease similarity. Next, the known association matrix and integrated similarity matrix are utilized to construct a probability matrix factorization algorithm to identify potentially relevant miRNAs for disease. We find that PMFMDA achieves reliable performance in the frameworks of global leave-one-out cross validation (LOOCV) and 5-fold cross validation (AUCs are 0.9237 and 0.9187, respectively) in the HMDD (V2.0) dataset, significantly outperforming a few state-of-the-art methods including CMFMDA, IMCMDA, NCPMDA, RLSMDA, and RWRMDA. In addition, case studies show that PMFMDA has good predictive performance for new associations, and the evidence can be identified by literature mining.

## Introduction

MicroRNAs are short non-coding RNAs. It plays a vital role in the regulation of many important biological processes (Bandyopadhyay et al., 2010; Hammond, 2015; Zhang et al., 2017). It has shown that human disease is associated with abnormal expression of miRNAs, whose analyses can guide the diagnosis, prognosis and treatment of certain diseases (Liang et al., 2019). However, identifying new miRNA–disease associations through bio-wet experiments not only has a high error rate, but also consumes huge financial resources (Feng et al., 2017). Therefore, *in-silicon* prediction of disease-associated miRNAs has become a critical step in prioritizing most confident targets for further experimental validation. Due to the growing power of sequencing technology, more and more omics data have been published (Yi et al., 2017), which provides a chance to reveal what role miRNAs play in physiology and pathology. Typical directions include miRNAs–disease interaction prediction, miRNA–miRNA regulatory module discovery, and so on (Chou et al., 2016). Undoubtedly, all these studies enrich our understanding of the functional regulation mechanisms of miRNA (Ha et al., 2019).

In recent years, in order to understand the pathogenesis of diseases, more and more computational models have been proposed by researchers to infer disease-related miRNAs, among which machine learning-based and network-based methods are most popular (Luo et al., 2017a). Network-based methods are based on a common assumption that miRNAs associated with diseases using similar phenotypes are similar in function, and vice versa. For example, Jiang et al. (2010) proposed the priority of disease-associated miRNAs through human peptide–microRNAome networks to identify potential associations. However, this method relies too much on known associations to make its prediction performance less effective. Subsequently, Chen et al. (2012) implemented a random walk with restart (RWRMDA) on its network to identify potentially associated miRNAs by building a network of similarities between miRNAs. Similarly, Shi et al. (2013) conducted random walks through functional linkages between miRNA targets and disease genes to explore the relationship between human miRNA diseases. Peng et al. (2017) constructed a multiple biological network by integrating the two-way relationship among microRNA, disease and environmental factors, and realized the unbalanced random walk algorithm on this network to achieve the purpose of prediction. However, these methods cannot predict miRNAs associated with isolated diseases. Later, Chen and Zhang (2013) used a network of consistent reasoning methods to infer unknown miRNAs associated with disease. Gu et al. (2016) created a network consistent projection algorithm to identify latent associations by integrating similarity networks and associated networks. The biggest advantage of these methods is that they can predict isolated disease-associated miRNAs, but the performance achieved is not very satisfactory.

More recently, machine learning-based models have been implemented to improve classification accuracy and prediction performance (Gu et al., 2016). For example, Xu et al. (2011) designed a support vector machine (SVM) classifier that combines four topological features extracted from a miRNA target disease network to distinguish between prostate cancer-associated miRNAs and non-prostate cancer-associated miRNAs. To construct a negative sample, they randomly paired the miRNA with the disease and then removed the pair present in the positive sample set. It is clear that negative samples constructed in this way are prone to false positives. Chen and Yan (2014) introduced a normalized least square method to identify the association between potential miRNAs–diseases (RLSMDA), which does not require negative samples. In addition, Luo et al. (2017b) developed a Kronecker regularized least squares method to predict the potential association of miRNAs–disease by combining multiple omics data. Liu et al. (2019) converted the miRNAs–disease association prediction problem into a complete bipartite graph model, and proposed a prediction algorithm based on a restricted Boltzmann machine to improve prediction performance. Shen et al. (2017) introduced the cooperative matrix decomposition (CMFMDA) algorithm in the recommendation system to infer potential associations. Finally, Chen et al. (2018) introduced an induction matrix-completed algorithm to identify unknown associations. However, these methods do not perform well in predicting associations related to new diseases or miRNAs, and the prediction accuracy is not as satisfactory as associations with known diseases or miRNAs.

In order to achieve better predictive performance, we construct a new model called probability matrix factorization (PMFMDA) to predict unknown miRNAs–disease associations in this study. PMFMDA makes full use of miRNA disease association, miRNA similarity and disease similarity. To evaluate the effectiveness of PMFMDA, we test it using frameworks of global 5-fold CV and global LOOCV. In addition, a validation method called *CV* *_d_* is developed to estimate the performance in predicting novel diseases or miRNAs. Outperforming other state-of-the-arts methods, PMFMDA achieve reliable performance in the frameworks of global LOOCV and 5-fold CV (AUCs of 0.9237 and 0.9187, respectively) in the HMDD (V2.0) dataset (Li et al., 2014). To further demonstrate the superiority of PMFMDA, we conduct an analysis of three common diseases. According to the analysis of the test results, we can find that there are 20, 19 and 17 of 20 candidate miRNAs that are confirmed to be associated with esophageal neoplasms, breast neoplasms and lung neoplasms by dbDEMC and miRCancer, respectively.

## Materials and Methods

**Figure 1:**
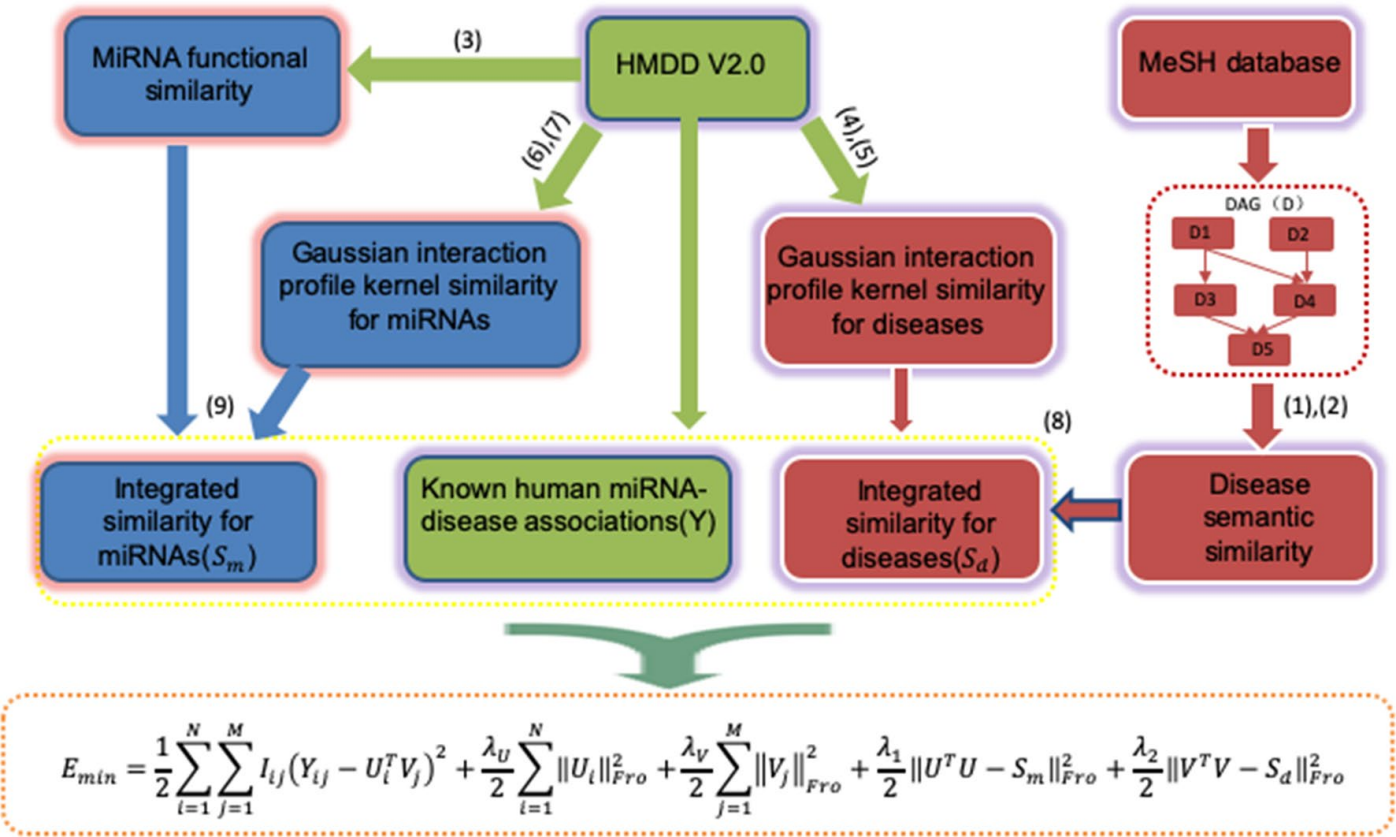
The workflow of PMFMDA is used to infer disease-associated unknown miRNAs.

The general workflow of PMFMDA is shown in **Figure 1**. We first use matrix Y to represent 5,430 experimentally validated associations after preprocessing the HMDD V2.0 database (Li et al., 2014). Specifically, Y is a 495 × 383 matrix with row denoting miRNAs and column denoting diseases; Y*_i,j_* = 1 if the *i* *^th^* miRNA is associated with the *j* *^th^* disease and 0 otherwise. We then calculate the disease similarity *S* *_d_* and miRNA similarity *S* *_m_*. Finally, a probability matrix factorization (PMF) model is proposed by integrating Y, *S* *_d_* and *S* *_m_*, the solution of which will recover unknown miRNAs–disease associations based on known ones.

### Disease Semantic Similarity

The hierarchical directed acyclic graphs (DAGs), usually are obtained from the MeSH database, and are widely used to calculate the similarity between diseases (Gu et al., 2016). Specifically, for a disease *d*, let *DAGd* = (*d*, *T* *_d_*, *E* *_d_*) represents its directed acyclic graph, where *T* *_d_* denotes the set of the ancestors of *d*, and *E* *_d_* represents the set of links in the MeSH tree. So, the semantic contribution of disease *t* to disease *d* is defined as:

(1) $$D_{d}\left(t\right)=\{\begin{array}{cc} 1 & if\;t=d\\ max\left\{∆\times D_{d}\left(t^{\prime }\right)|t^{\prime }\in children\;of\;t\right\} & if\;t\neq d \end{array}$$

Where Δ is a predefined sematic contribution factor, the value of Δ in this study is set to 0.5. Therefore, we can calculate the semantic similarity of between diseases by formula (2).

(2) $$D(d_{i},d_{j})=\frac{\sum_{t\in T_{d_{i}}\cap T_{d_{j}}}\left(D_{d_{i}}(t)+D_{d_{j}}(t)\right)}{\sum_{t\in T_{d_{i}}}D_{d_{i}}(t)+\sum_{t\in T_{d_{j}}}D_{d_{j}}(t)}\;\;\;\;\;\;\;\;\;\;\;\;\;\;\;\;\;$$

### miRNAs Functional Similarity

For the similarity between miRNAs, most studies use functional similarity measurements (Wang et al., 2010). Specifically, for any two miRNAs *r* *_i_* and *r* *_j_*, let *DT* *_i_* = {*d* *_i_*_1_,*d* *_i_*_2_,…,*d* *_i_*_k_} and *DT* *_j_* = {*d* *_j_*_1_,*d* *_j_*_2_,…,*d* *_jl_*} be their associated disease sets, respectively. Similar to Wang et al. we first use $S(d,\;DT)=\overset{max}{d_{i}\in DT}D(d,d_{i})$ to represent the similarity between a disease *d* and *DT*. Then the similarity between *r* *_i_* and *r* *_j_* is defined as

(3) $$R(r_{i},r_{j})=\frac{\sum_{m=1}^{k}S(d_{im},DT_{j})+\sum_{n=1}^{l}S(d_{jn},DT_{i})}{k+l}$$

### The Gaussian Interaction Profile Kernel Similarity For Diseases and miRNAs

In the similarity measurement algorithm, Gaussian interaction profile kernel similarity is also a good measurement algorithm, which is widely used in various fields (Lu et al., 2019). Let *VP*(*d* *_i_*) be the vector associated with the disease *d* *_i_* in Y, i.e. the *i* *^th^* column of Y. Then, the Gaussian interaction kernel similarity between disease *d* *_i_* and *d* *_j_* is calculated as:

(4) $$KD(d_{i}\;,d_{j})=exp(-\gamma_{d}||VP(d_{i})-VP(d_{j})|{|}^{2})$$

where γ*_d_* is the adjustment parameter of the kernel bandwidth. The parameter γ*_d_* update rule is as follows:

(5) $$\gamma_{d}=\gamma_{d}^{'}/(\frac{1}{nd}\sum_{i=1}^{nd}||(d_{i})|{|}^{2})$$

where $\gamma_{d}^{'}$ is usually set to 1.

Similarly, we can conclude that the Gaussian kernel similarity of miRNAs is as follows:

(6) $$KM\left(r_{i}\;,r_{j}\right)=exp(-\gamma_{m}||VP(r_{i})-VP(r_{j})|{|}^{2})$$

(7) $$\gamma_{m}=\gamma_{m}^{'}/(\frac{1}{nm}\sum_{i=1}^{nm}||VP(r_{i})|{|}^{2})\;$$

Where $\gamma_{m}^{'}$ is usually set to 1.

### Integrated Similarity For Diseases and miRNAs

The similarity between disease *d* *_i_* and disease *d* *_j_* is constructed by combining the two similarities of the disease as follows:

(8) $$S_{d}(d_{i}\;,d_{j})=\{\begin{array}{cc} D(d_{i}\;,\;d_{j}) & \;\;d_{i}\;and\;d_{j}\;has\;semantic\;similarity\\ KD(d_{i}\;,d_{j}) & otherwise \end{array}$$

Similarly, the similarity between miRNAs *r* *_i_* and *r* *_j_* can be redefined as:

(9) $$S_{m}(r_{i}\;,r_{j})=\{\begin{array}{cc} R(r_{i}\;,r_{j}) & \;\;\;r_{i}\;and\;r_{j}\;has\;functional\;similarity\\ KM(r_{i}\;,r_{j}) & otherwise \end{array}$$

## PMFMDA

Probability Matrix Decomposition (PMF) is a probabilistic linear model of Gaussian observation noise and has been widely used in data representation (Salakhutdinov and Mnih, 2008). Let *Y*∈*R* *^n×m^* be the known miRNAs–disease association matrix, *U* *_i_* and *V* *_i_* represent the D-dimensional miRNA-specific and disease-specific latent feature vectors, respectively. The conditional distribution of the observed associations *Y*∈*R* *^n×m^* (likelihood term) and the prior distribution of *U*∈*R* *^D×n^* and *V*∈*R* *^D×m^* are given by:

(10) $$P\left(Y|U,V,\alpha \right)=\prod_{i=1}^{N}\prod_{j=1}^{M}{\left[N(Y_{ij}|U_{i}^{T}V_{j},\alpha^{-1})\right]}^{I_{ij}}$$

(11) $$P(U|\alpha_{U})=\prod_{i-1}^{N}N(U_{i}|0,\alpha_{U}^{-1}I)$$

(12) $$P(V|\alpha_{V})=\prod_{j=1}^{M}N(V_{j}|0,\alpha_{V}^{-1}I)$$

Where *N* (*x* | *µ*,α^-1^) denotes the Gaussian distribution, *I* *_ij_* = 0 if the entry(*i,j*) in *Y* is missing, and 1 otherwise.

The optimal model is obtained by maximizing the logarithmic a posterior of miRNAs and disease characteristics using fixed hyperparameters:

(13) $$\ln P\left(U,V|Y,\alpha ,\alpha_{V},\alpha_{U}\right)=\ln P\left(Y|U,V,\alpha \right)+\ln P\left(U|\alpha_{U}\right)+\ln P\left(V|\alpha_{V}\right)+C$$

Where C is a constant. So, using a quadratic regularization term to minimize the sum of squares of the error functions instead of maximizing the posterior distribution relative to U and V:

(14) $$E_{\min}=\frac{1}{2}\sum_{i=1}^{N}\sum_{j=1}^{M}I_{ij}{\left(Y_{ij}-U_{i}^{T}V_{j}\right)}^{2}+\frac{\lambda_{U}}{2}\sum_{i=1}^{N}||U_{i}|{|}_{Fro}^{2}+\frac{\lambda_{V}}{2}\sum_{j=1}^{M}||V_{j}|{|}_{Fro}^{2}$$

Where *λ* *_U_* = *α* *_U_* / *α* and *λ* *_v_* = *α* *_V_* / *α* are regularization parameters, $||\cdot |{|}_{Fro}^{2}$ denotes the Frobenius norm.

The standard PMF in Equation (10) does not consider the effect of similarity between miRNAs and the similarity between diseases. Since *U* *_i_* represents the D-dimensional miRNA-specific latent feature vectors, *U* *^T^* *U* denotes the weighted similarity matrix of the miRNAs. Similarly, *V* *^T^* *V* denotes the weighted similarity matrix of the disease. Thus, we propose a new objective function by integrating miRNAs similarity and diseases similarity named PMFMDA as follows:

(15) $$E_{\min}=\frac{1}{2}\sum_{i=1}^{N}\sum_{j=1}^{M}I_{ij}{\left(Y_{ij}-U_{i}^{T}V_{j}\right)}^{2}+\frac{\lambda_{U}}{2}\sum_{i=1}^{N}||U_{i}|{|}_{Fro}^{2}+\frac{\lambda_{V}}{2}\sum_{j=1}^{M}||V_{j}|{|}_{Fro}^{2}+\frac{\lambda_{1}}{2}||U^{T}U-S_{m}|{|}_{Fro}^{2}+\frac{\lambda_{2}}{2}||V^{T}V-S_{d}|{|}_{Fro}^{2}$$

where *S* *_m_* and *S* *_d_* have been calculated before.

### Optimization

In order to obtain the local optimal solution of Equation (15), we use the gradient descent algorithm to solve (Xiao et al., 2018). According to the nature of the Frobenius norm, the corresponding Lagrange function *L* *_E_* of Equation (15) is defined as:

(16) $$L_{E}=\frac{1}{2}Tr\left(I\cdot (YY^{T}-YV^{T}U-U^{T}VY^{T}+U^{T}VV^{T}U)\right)+\frac{\lambda_{U}}{2}Tr(UU^{T})+\frac{\lambda_{V}}{2}Tr(VV^{T})+\frac{\lambda_{1}}{2}Tr(S_{m}{\left(S_{m}\right)}^{T})-S_{m}U^{T}U-U^{T}U(S_{m})+U^{T}UU^{T}U)+\frac{\lambda_{2}}{2}Tr(S_{d}{\left(S_{d}\right)}^{T}-S_{d}V^{T}V-V^{T}VS_{d}+VV^{T}V)+Tr(\emptyset U^{T})+Tr(\psi V^{T})$$

where *T* *_r_*() denotes the trace of a matrix, ∅=[ϕ*_ik_*] and Ψ=[ω*_jk_*] are Lagrangian multipliers.

The partial derivatives of *U* and *V* are as follows:

(17) $$\begin{array}{l} \frac{\partial L_{E}}{\partial U}=I\cdot \left(-VY^{T}+VV^{T}U\right)+\lambda_{U}U+2\lambda_{1}\left(-U\left(S_{m}\right)+UU^{T}U\right)+\emptyset ,\\ \frac{\partial L_{E}}{\partial V}=I\cdot \left(-UY+UU^{T}V\right)+\lambda_{V}V+2\lambda_{2}\left(-V\left(S_{d}\right)+VV^{T}V\right)+\Psi \end{array}$$

Finally, the Karush-Kuhn-Tucker (KKT) conditions $\varphi_{ikU_{ik}=0}$ and $\omega_{jkV_{Jk}=0}$ according to the gradient descent method. The following equations are obtained for *U* *_ik_* and *V* *_jk_*:

(18) $${\left(I\cdot \left(-VY^{T}+VV^{T}U\right)\right)}_{ik}U_{ik}+{\left(\lambda_{U}U\right)}_{ik}U_{ik}+{\left(2\lambda_{1}\left(-U\left(S_{m}\right)+UU^{T}U\right)\right)}_{ik}U_{ik}=0,{\left(I\cdot (-UY+UU^{T}V)\right)}_{jk}V_{jk}+{\left(\lambda_{V}V\right)}_{jk}V_{jk}+{\left(2\lambda_{2}(-V(S_{d})+VV^{T}V)\right)}_{jk}V_{jk}=0$$

Therefore, the updating rules for *U* and *V* as follows:

(19) $$\;U_{ik}^{new}=U_{ik}\frac{{\left(I\cdot (VY^{T})+2\lambda_{1}(U(S_{m}))\right)}_{ik}}{{\left(I\cdot (VV^{T}U)\right)}_{ik}+{\left(\lambda_{U}U\right)}_{ik}+{\left(2\lambda_{1}(UU^{T}U)\right)}_{ik}}$$

(20) $$V_{jk}^{new}=V_{jk}\frac{{\left(I\cdot (UY)+2\lambda_{1}(U(S_{m}))\right)}_{jk}}{{\left(I\cdot (UU^{T}V)\right)}_{jk}+{\left(\lambda_{V}V\right)}_{jk}+{\left(2\lambda_{2}(VV^{T}V)\right)}_{jk}}$$

Update *U* and *V* according to Equation (19) and Equation (20) until the local minimum of the objective function. Finally, the predicted miRNAs–disease association matrix is *Y* *^′^*=*U* *^T^* *V*. The *i*th column of *Y*′ indicates the association score between disease *d* *_i_* and miRNAs, and the larger the score, the more relevant it is.

### Evaluation Methods

In order to test the performance of PMFMDA, we utilize a 5-fold CV experiment and global LOOCV on the HMDD database and compare it with a few recent methods including CMFMDA, IMCMDA, NCPMDA, RLSMDA, and RWRMDA. In the 5-fold CV experiment of a single disease *d*, known miRNAs associated with *d* (column vectors in matrix A∈R^m×n^) are randomly divided into five subsets of equal size. Associations related to all other diseases together with 4 subsets (with respect to *d*) are taken as training samples and the remaining subset is considered as testing samples. The process is performed for 5 times until all the associations associated with d have been predicted once. Global LOOCV was used to evaluate the model’s global prediction ability for all miRNAs–disease association simultaneously. Specifically, we removed each known association in turn as a testing sample, with all remaining associations as training samples. We then predicted the removed entry and evaluated the performance. In addition, we perform *CV* *_d_* experiment to test the performance of PMFMDA in predicting miRNAs associated to a novel disease *d.* In *CV* *_d_*: CV on disease *d* *_i_*, we remove all the known associations of the disease *d* *_i_* (column vectors in matrix *Y*∈R^m×n^) and build prediction model (for inferring the deleted associations) using the remaining data.

### Parameter Tuning

We cross-validate the training set to tune the parameters of PMFMDA. Specifically, the parameters *λ* *_U_*,*λ* *_V_*,*λ*_1_, and *λ*_2_ are increased from 0.001 to 1 with a step of 0.1 and the ones with the best AUC are selected. Since the other methods have also been tested on HMDD (V2.0) in published papers, we adopt the parameters provided by the authors. Specifically, *W*=0.9 for RLSMDA, *λ* *_U_* = *λ* *_V_* = 1,*λ*_1_ = *λ*_2_ = 0.005 for PMFMDA, *λ*_1_ = *λ*_2_ = 1 for IMCMDA, *λ* *_m_* = *λ* *_d_* = 1 for CMFMDA *r* = 0.9, for RWRMDA and NCPMDA is parameter free.

## Results

### PMFMDA Outperforms Other Popular Methods In Predicting Potential Associations

**Figure 2:**
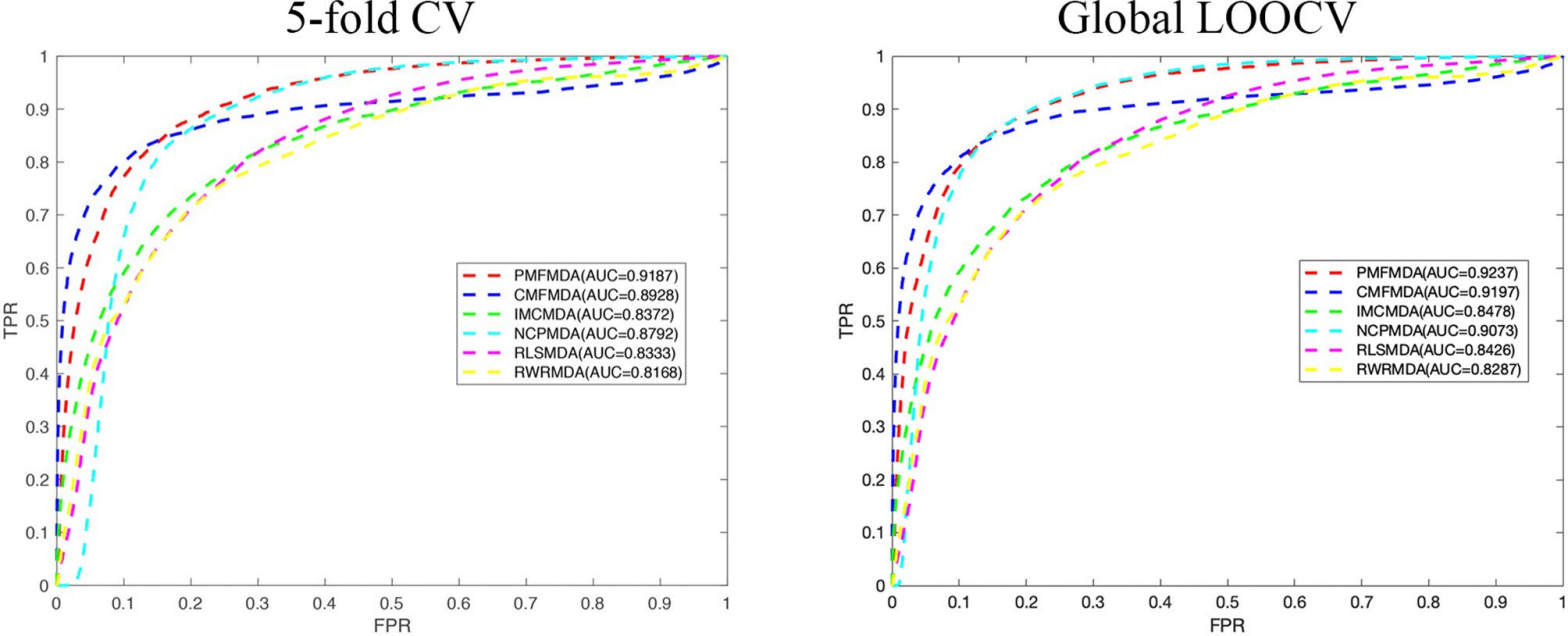
The ROC curves for PMFMDA and benchmark algorithms for 5-fold CV and global LOOCV.

**Figure 3:**
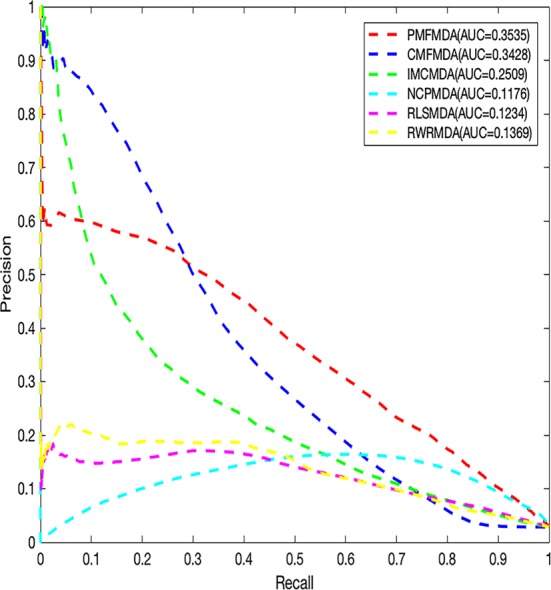
The PR curves for PMFMDA and benchmark algorithms for 5-fold CV.

We apply PMFMDA, CMFMDA, IMCMDA, NCPMDA, RLSMDA, and RWRMDA into the HMDD database. Their receiver operating characteristic (ROC) curves and associated area under the curve (AUCs) of the global 5-fold CV and LOOCV are plotted in **Figure 2**. As can be seen, the AUCs of PMFMDA, CMFMDA, IMCMDA, NCPMDA, RLSMDA, and RWRMDA are 0.9187, 0.8928, 0.8372, 0.8792, 0.8333, and 0.8168, respectively. Furthermore, PMFMDA also achieve the best AUC (0.9237) on global LOOCV, indicating that PMFMDA perform best in predicting miRNAs–disease associations. However, considering the limited number of known miRNAs–disease associations, it might be insufficient to evaluate the performance of the methods by AUC alone. Thus, we also plotted the precise recall (PR) curve and calculated the area under the PR curve (AUPR) based on the global 5-fold CV experiment in **Figure 3**. In a PR-curve, the precision refers to the ratio of correctly predicted associations to all associations with scores higher than a given threshold; by contrast, the recall refers to the ratio of correctly predicted associations to all known miRNAs–disease associations. In general, the ROC curve and the PR curve show similar trend. As shown in **Figure 3**, the AUPRs of PMFMDA, CMFMDA, IMCMDA, NCPMDA, RLSMDA, and RWRMDA are 0.3535, 0.3428, 0.2509, 0.1176, 0.1234, and 0.1369 respectively, indicating that PMFMDA performed best in predicting miRNAs–disease associations. At the same time, in order to further prove the effectiveness of PMFMDA. We performed 10 times of global 5-fold CV and achieved an average AUC and AUPR of 0.9187 +/− 0.0013, 0.3535+/− 0.0015, respectively. This proves the reliability and stability of the PMFMDA algorithm.

### PMFMDA Outperforms Other Popular Methods In Predicting miRNAs Associated With Novel Diseases

Besides global miRNAs–disease predictions, it is also critical to check the performance of the above methods on specific diseases. *CV* *_d_* is used to measure the ability of an algorithm to predict a new disease-associated miRNA. In order to compare the fairness of the test, we conduct CV tests on 8 common diseases (Xuan et al., 2015) and use the area under the accurate recall curve (AUPR) as an indicator of predictive performance. The reason is that AUPR severely penalizes highly ranked non-interactions, which is desirable here because in practice we do not want to recommend incorrect predictions (i.e., AUPR metrics severely penalize highly ranked false positives). The results for *CV* *_d_* are shown in **Table 1**. We can clearly see that the average AUPR of PMFMDA for the eight test diseases was 0.6687, which was significantly higher than IMCMDA (0.6377), CMFMDA (0.5091), NCPMDA (0.6121), and RLSMDA (0.5761). This also sufficient PMFMDA is also the best way to predict miRNAs associated with novel diseases.

**Table 1:** Comparison of AUPR values predicted by PMFMDA and benchmark algorithms on novel diseases.

Disease name	AURP				
	PMFMDA	IMCMDA	CMFMDA	NCPMDA	RLSMDA
Melanoma	0.7149	0.6757	0.4574	0.6785	0.6940
Breast tumor	0.7895	0.7752	0.6135	0.7866	0.7749
Colorectal tumor	0.6585	0.6333	0.4725	0.5714	0.5315
Glioblastoma	0.5940	0.5076	0.4540	0.4779	0.4028
Heart failure	0.5956	0.6284	0.4510	0.6182	0.5510
Prostatic tumor	0.6578	0.5881	0.5963	0.5873	0.5208
Stomach tumor	0.6981	0.6438	0.5231	0.6269	0.6081
Bladder tumor	0.6409	0.5388	0.5051	0.5505	0.5255
Mean	0.6687	0.6237	0.5091	0.6121	0.5761

Furthermore, in order to further evaluate our approach in predicting new diseases. We implement *CV* *_d_* experiments on the above 8 diseases. We show the calculation of the number of disease-associated miRNAs identified at different ranking thresholds in **Table 2**. For example: We delete all miRNAs associated with breast tumors, and then use PMFMDA to predict its related miRNAs. we can find that 91 of the top 100 predictions are accurately predicted through the test results. This is ample indication that our approach can yield high quality predictions for isolated disease-associated miRNAs. In order to better understand the predicted eight disease-related miRNAs, we listed the names and predicted scores of the top 100 candidates related to the eight diseases in the **Supplementary Table S1**.

**Table 2:** PMFMDA predicts the correct numbers of different ranking thresholds for 8 common diseases.

Cancer	No. of known associated miRNAs	Ranking threshold				
		20	40	60	80	100
Breast neoplasms	202	20	38	54	74	91
Colorectal neoplasms	147	17	30	45	58	70
Glioblastoma	96	17	30	36	43	53
Heart failure	120	17	28	39	51	58
Melanoma	141	19	35	51	63	77
Prostatic neoplasms	118	17	32	43	56	65
Stomach neoplasms	173	15	32	49	63	79
Urinary bladder neoplasms	92	18	31	42	51	55

### Evaluate Performance on Different Data Sources

To further test the versatility of PMFMDA. We obtain 60,576 experimental validation correlation data by preprocessing the MNDR (V2.0) dataset (Cui et al., 2018). The data contains 887 diseases and 3,954 miRNAs. We apply PMFMDA, CMFMDA, IMCMDA, NCPMDA, RLSMDA, and RWRMDA on the MNDR (V2.0) database. As shown in **Table 3**, the AUC of PMFMDA was 0.9885, significantly higher than those of CMFMDA (0.9799), IMCMDA (0.9171), NCPMDA (0.9480), RLSMDA (0.9358), and RWRMDA (0.9055) with increases of about 0.86, 7.14, 4.05, 5.27, and 8.3% respectively. The AUPR of PMFMDA was 0.5174, significantly higher than those of CMFMDA (0.5047), IMCMDA (0.3865), NCPMDA (0.2045), RLSMDA (0.2818), and RWRMDA (0.1907). In conclusion, PMFDA has been proven to be effective in inferring related miRNAs with diseases in terms of AUC values and AUPR values.

**Table 3:** The performance of PMFMDA and the baseline methods based on 5-fold CV on the MNDRV2.0 dataset.

	PMFMDA	CMFMDA	IMCMDA	NCPMDA	RLSMDA	RWRMDA
AUC	0.9885	0.9799	0.9171	0.9480	0.9358	0.9055
AUPR	0.5174	0.5047	0.3865	0.2045	0.2818	0.1907

### Parameter Sensitivity Analysis

In machine learning, parameter tuning is critical for the performance of a model. Thus, we presented in **Table 4** several sets of parameter settings based on the global 5-fold CV experiment on the HMDDV 2.0 dataset. We found that a better prediction result will be achieved when the value of *λ*_1_ and *λ*_2_ are large and the value of *λ*_1_ and *λ*_2_ are small. This result further confirms the effectiveness of seeking an optimal combination of parameters in improving performance.

**Table 4:** Parameter tuning for PMFMDA based on 5-fold CV.

AUC	*λ*_U_ = *λ*_V_ = 1	*λ*_U_ = *λ*_V_ = 0.1	*λ*_U_ = *λ*_V_ = 0.01
*λ*_1_ = *λ*_2_ = 1	0.7905	0.7728	0.7588
*λ*_1_ = *λ*_2_ = 0.1	0.9040	0.8507	0.8381
*λ*_1_ = *λ*_2_ = 0.01	0.9185	0.9032	0.8692

**Figure 4:**
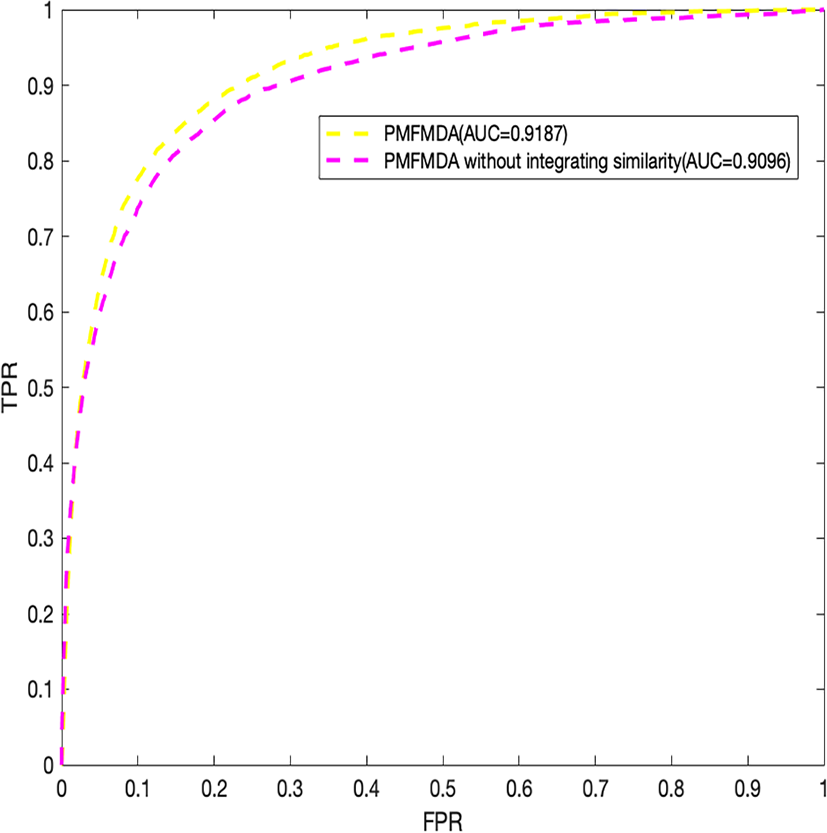
Performance evaluation of PMFMDA in two situations for 5-fold cross validation. (1) PMFMDA with similarity information; (2) PMFMDA without similarity information.

Finally, we explore the effect of the disease similarity and miRNA similarity on prediction performance. Specifically, we perform global 5-fold CV with parameters *λ*_1_ and *λ*_2_ setting to zero (**Figure 4**) in the HMDD (V2.0) dataset. We can see that the two similarities do contribute to prediction performance. In addition, PMFMDA achieve good results even in the model without integrating disease and miRNA similarity. However, this model is not good in predicting the association of new diseases or new miRNAs.

### Case Studies

Another aspect of PMFMDA’s strong predictive power is in case studies. Here, all the associations included in the HMDD (V2.0) database are used as training for the model, and the unincorporated associations are considered candidates for verification. In addition, miRCancer (Xie et al., 2013) and dbDEMC (Yang et al., 2010) were used to verify the correctness of the predictions. In this work, we mainly study three diseases including esophageal tumors, breast tumors, and lung tumors, and perform detailed analyses of the top 10 candidates predicted by PMFMDA in each disease (see **Table 5**).

**Table 5:** PMFMDA infers the top 10 miRNA candidates for the three selected diseases.

Cancer	Number of miRNAs identified by the literature	Top 10					
		Rank	miRNAs	Evidence	Rank	miRNAs	Evidence
Esophageal neoplasms		1	mir-17	dbDEMC	6	mir-1	dbDEMC
		2	mir-18a	dbDEMC	7	mir-200b	dbDEMC
	10	3	mir-221	dbDEMC	8	mir-222	dbDEMC
		4	mir-16	dbDEMC	9	mir-29a	dbDEMC
		5	mir-19b	dbDEMC	10	mir-133b	dbDEMC
Breast neoplasms		1	mir-142	miRCancer	6	mir-138	dbDEMC
		2	mir-150	dbDEMC, miRCancer	7	mir-15b	dbDEMC
	9	3	mir-106a	dbDEMC	8	mir-192	dbDEMC
		4	mir-99a	dbDEMC, miRCancer	9	mir-378a	Unconfirmed
		5	mir-130a	dbDEMC	10	mir-196b	dbDEMC
lung neoplasms		1	mir-16	dbDEMC	6	mir-99a	dbDEMC
		2	hsa-mir-15a	dbDEMC	7	mir-429	dbDEMC, miRCancer
	9	3	hsa-mir-106b	dbDEMC	8	mir-302b	dbDEMC, miRCancer
		4	mir-195	dbDEMC, miRCancer	9	mir-130a	dbDEMC
		5	mir-141	dbDEMC	10	mir-296	Unconfirmed

Esophageal tumors are a disease with high morbidity and high mortality in the digestive system (Kano et al., 2010; He et al., 2012). Early diagnosis plays a crucial role in its treatment (Azmi, 2012). In this study, we use PMFMDA to identify potential miRNAs associated with esophageal tumors. The top 10 miRNAs to be all confirmed by the database were associated with esophageal tumors (see **Table 5**).

Breast neoplasm is the malignant tumor that is prone to occur in women, it is a systemic malignant disease, for which many related genes have been discovered (Venkatadri et al., 2016). MicroRNA (miRNA), as a kind of small RNA, can specifically bind to the 3′ untranslated region of its target mRNA, causing translational inhibition or degradation of target mRNA, and playing an oncogene in the process of cell growth and differentiation (Miller et al., 2008). Thus, MiRNAs present a new way for the study of pathogenic genes in breast neoplasms. As we can see from **Table 5**, 9 of the top 10 predictions have been confirmed by the relevant databases.

The death rate from lung neoplasms is extremely high. About 1.3 million people die of lung neoplasms every year, accounting for about one-third of all neoplasms deaths worldwide (Yu et al., 2015; Sun et al., 2016). miRNAs have been found as a tumor suppressor gene and lung neoplasms. For example, Gu et al. found that miR-99a was significantly expressed in lung cancer tissues and lung neoplasm cells. In addition, the expression level of miR-99a is correlated with clinicopathological factors, the clinical stage and lymph node metastasis of lung cancer patients. We use PMFMDA to predict potential related miRNAs in lung tumors. As shown in **Table 5**, we can find that only one of the top 10 related miRNAs predicted is unconfirmed.

**Figure 5:**
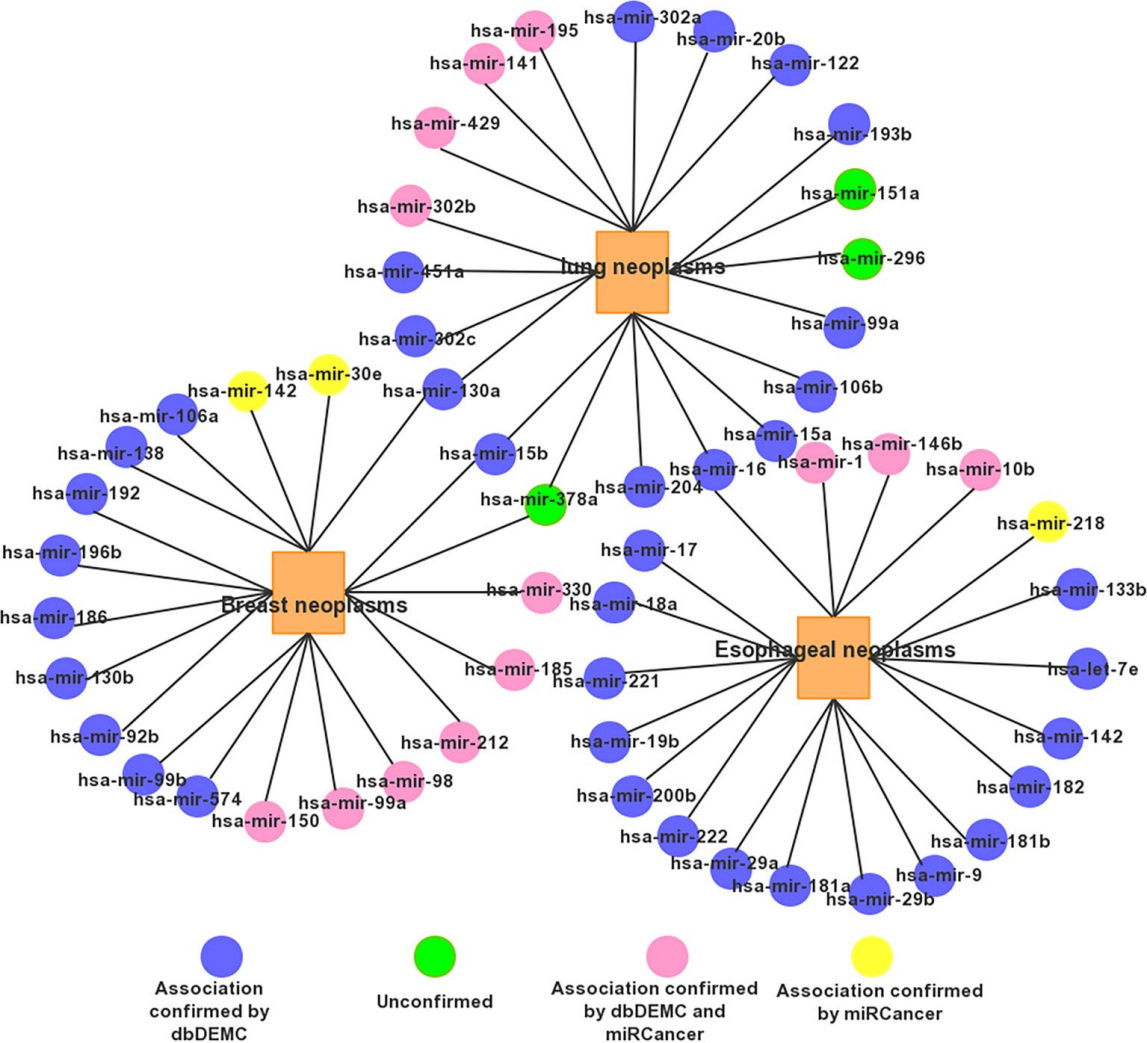
The network of the top 20 predicted associations for the three selected diseases via PMFMDA.

For a clear view, we show the top 20 miRNAs associated networks predicting three tumors in **Figure 5**. It is worth noting that some miRNA candidates are usually associated with several diseases. For example, mir-15b and mir-130a are associated with both Prostatic lung and Breast Neoplasms. Has-mir-16 is associated with both Esophageal Neoplasms and lung Neoplasms.

## Discussion

It is known that miRNAs often play an irreplaceable role in biological processes related to human diseases (Shen et al., 2017). Accurately inferring disease-related potential miRNAs is helpful for us to investigate the pathogenesis of the disease and find a more effective treatment. In this study, we construct a mathematical model based on probability matrix factorization (PMFMDA) to identifying potential miRNAs–disease associations. PMFMDA outperform a few state-of-the-art models in the HMDD V2.0 database due to a few factors. First, PMFMDA not only uses known correlation data, but also integrates the similarities between miRNAs and between diseases. This has enabled PMFMDA to achieve good results in predicting isolated disease-associated miRNAs since theoretically similar miRNAs may associate with similar diseases. Second, the model is a semi-supervised model, which does not rely on negative samples. Thus, it is better than most machine learning algorithms with strong requirement for good negative samples. Finally, in the model solving process, we use the alternating gradient descent algorithm to find the optimal solution to ensure the reliability of disease feature vectors and miRNA feature vectors. In terms of experiment, PMFMDA achieves the highest AUC (0.9187, 0.9237, respectively) in 5-fold CV and global LOOCV, demonstrates its most reliable prediction performances. At the same time, we also perform *CV* *_d_* experiments to measure the ability of PMFMDA to predict miRNAs associated with novel diseases. We conduct CV testing on 8 common diseases, which have at least 80 associations are verified (Xuan et al., 2015). PMFMDA achieves the highest average AUPRs of 0.6687. Finally, to make the more comprehensive test of PMFMDA, we use the three most common diseases in humans for research. The number of other database validations in the top 20 predicted miRNAs for esophageal tumors, breast tumors, and lung tumors are found to be 20, 19, and 17, respectively. In conclusion, PMFMDA has achieved good results in predicting the potential association of miRNA disease and predicting new disease-associated miRNAs and can be used as a very useful supplement to existing prediction models.

Although quite satisfactory results have been achieved from PMFMDA, there are still some limitations to this approach. Firstly, we only use semantic similarity and the Gaussian kernel similarity to construct disease similarity network. It may be helpful to improve the predictive performance of PMFMDA by integrating disease or miRNA similarity from multiple data sources such sequence similarity. Secondly, the public data sets used in this study may have noise and outliers. A preprocessing step for de-noising and dimension reduction in raw input data might be useful. Thirdly, in the process of solving PMFMDA, the gradient descent method often obtains the local optimal solution, and how to further optimize its solution helps to improve the prediction performance of PMFMDA. Finally, as more and more miRNAs and disease associations are confirmed, collecting more validated data will help us to conduct more in-depth research.

## Data Availability Statement

The program and data used in this study are publicly available at: https://github.com/xujunlin123/PMFMDA.git.

## Author Contributions

JY, JX, LC, GT and BL conceived the concept of the work. JX, PW, WZ, YM, and JL performed the experiments. JX and JY wrote the paper. GT helped in revising the manuscript.

## Funding

This study is supported by National Nature Science Foundation of China (Grant Nos. 11171369, 61272395, 61370171, 61300128, 61472127, 61572178, 61672214, and 61772192), and the Natural Science Foundation of Hunan, China (Grant Nos. 2018JJ2461, 2018JJ3570).

## Conflict of Interest

The authors JL and GT were employed by Geneis Beijing Co., Ltd.

The remaining authors declare that the research was conducted in the absence of any commercial or financial relationships that could be construed as a potential conflict of interest.
